# Characterization of Structural and Physicochemical Properties of an Exopolysaccharide Produced by *Enterococcus* sp. F2 From Fermented Soya Beans

**DOI:** 10.3389/fmicb.2021.744007

**Published:** 2021-10-29

**Authors:** Guangyang Jiang, Longzhan Gan, Xiaoguang Li, Juan He, Shihao Zhang, Jia Chen, Ruoshi Zhang, Zhe Xu, Yongqiang Tian

**Affiliations:** ^1^College of Biomass Science and Engineering, Sichuan University, Chengdu, China; ^2^Key Laboratory of Leather Chemistry and Engineering, Sichuan University, Ministry of Education, Chengdu, China; ^3^Key Laboratory of Bio-Resources and Eco-Environment of Ministry of Education, College of Life Sciences, Sichuan University, Chengdu, China

**Keywords:** exopolysaccharide, structure characterization, rheological properties, *Enterococcus* sp., functional properties

## Abstract

The present study sought to isolate a novel exopolysaccharide (EPS-F2) from *Enterococcus* sp. F2 through ethanol precipitation, anion-exchange, and gel-filtration chromatography and characterize the physicochemical properties by spectral techniques. EPS-F2 was identified as a neutral homo-exopolysaccharide composed of only glucose with a high molecular weight of 1.108 × 10^8^ g/mol. It contained →6)-α-D-Glc*p*-(1→ linkage in the main chain and →3, 6)-α-D-Glc*p*-(1→ branch chain). Moreover, EPS-F2 possessed excellent thermal stability (266.6°C), water holding capacity (882.5%), oil holding capacity (1867.76%), and emulsifying activity against various edible oils. The steady shear experiments exhibited stable pseudo plasticity under various conditions (concentrations, temperatures, and pHs). The dynamic oscillatory measurements revealed that EPS-F2 showed a liquid-like behavior at a low concentration (2.5%), while a solid-like behavior at high concentrations (3.0 and 3.5%). Overall, these results suggest that EPS-F2 could be a potential alternative source of functional additives and ingredients and be applied in food industries.

## Highlights

- A homopolysaccharide (EPS-F2) with high Mw was obtained from *Enterococcus* sp. F2.
- It had a noteworthy thermal stability under high temperature.
- Functional properties of EPS-F2 from *Enterococcus* sp. F2 were assessed.
- The non-Newtonian pseudoplastic behavior was observed in EPS-F2 solutions.

## Introduction

Exopolysaccharides (EPSs) are high molecular weight carbohydrate polymers secreted to the extracellular matrix by various microorganisms. EPSs are classified into two major groups based on their monosaccharide compositions: (1) homo-exopolysaccharides, comprising of a single type of sugar with repeating subunits; (2) hetero-exopolysaccharides, comprising of two and more types of monosaccharide subunits (Riaz Rajoka et al., 2020). EPSs have significant advantages over other plant polysaccharides due to their seasonal independence and geographical conditions. Unlike synthetic polymers, the naturally occurring EPSs are environmentally safe with excellent biodegradability and biocompatibility. These distinctive properties have increased the application of EPSs in various industries, such as food, pharmaceutical, textile, cosmetics, and chemical products (Hu et al., 2019). EPS structure and properties vary depending on their species, media ingredients, and culture conditions. Furthermore, their functional and rheological properties also vary in different industries, due to their glycosidic bonds, molecular weights, and functional groups.

Exopolysaccharide obtained from different *Lactic acid bacteria* (*LAB*) strains have attracted wide attention in the past decades due to their structural diversity and functional properties. Accumulating studies have described the structural characteristics, functional and rheological properties, and potential bioactivity of EPS produced by *lactobacilli* (Zhou et al., 2019), *Weissella cibaria* (Aburas et al., 2020), and *Lactobacillus plantarum* (Wang et al., 2015). Fermented soya beans, one of the most popular fermented foods in China, possess rich microbial resources, such as *Enterobacter, Enterococcus, Leuconostoc*, and *Lactobacillus* (Xie et al., 2019, 2020). *Enterococcus*, a type of lactic acid bacteria found in various fermented foods and in the gut of humans or animals, are generally regarded as safe (GRAS) bacteria (Bhat and Bajaj, 2018; Jia et al., 2019). *Enterococcus* also plays a vital role in the development of fermented food products by promoting their aromas and flavors (Yoon et al., 2008). Previous studies on the safety of *Enterococcus* isolated from different fermented foods have indicated that this bacteria might exert beneficial probiotic effects and antagonistic activities (Liu et al., 2015). Similarly, few other studies have investigated the properties of *Enterococcus* induced EPS isolated from processed fish products (Abdhul et al., 2014), fermented milk product (Bhat and Bajaj, 2018), and human breast milk (Kansandee et al., 2019).

However, the structural characteristics and physicochemical properties (functional properties and rheological properties) of the screened EPS were not elucidated by these studies. Therefore, this study aimed to isolate, purify, and characterize EPS-F2, and analyze its functional properties, including emulsifying activity (EA), oil holding capacity (OHC), water solubility index (WSI), and water holding capacity (WHC). Furthermore, the rheological properties influenced by concentrations, temperatures, pH values, and salts were assessed.

## Materials and Methods

### Screening and Identification of Exopolysaccharide-Producing Lactic Acid Bacteria Strain

The EPS-producing strain F2 was isolated from Guizhou fermented soya beans from Liupanshui city, Guizhou province, China. Fermented soya bean samples were homogenized and serially diluted in sterile distilled water. Each diluted sample was spread on an agar plate containing MRS medium and incubated at 30°C until single colonies appeared. The strain F2 was identified by cell morphological and 16S rDNA sequence analyses (Galli et al., 2020). The phylogenetic tree was constructed by MEGA 7.0 using the neighbor-joining method.

### Preparation and Purification of Crude Exopolysaccharide

For EPS production, strain F2 was cultured in fresh MRS broth containing 40 g/L sucrose at 30°C for 48 h. Crude EPS were extracted from the strain and purified according to a previously reported method (Ayyash et al., 2020b), with minor modifications. Briefly, the cells were harvested by centrifugation at 7000 × *g* at 4°C for 20 min. The supernatant was mixed with three pre-chilled 95% (v/v) ethanol volumes to precipitate the crude EPS. The resultant crude EPS was treated with 8% (w/v) trichloroacetic acid to remove the precipitated proteins. After centrifugation, the obtained solution was dialyzed with ultrapure water using a dialysis bag (Mw cut off = 8000–14,000 Da) for 48 h and, finally, lyophilized.

The lyophilized EPS was resuspended in ultrapure water and filtered through a 0.45 μm syringe filter. The filtrate was fractionated through a DEAE-52 anion-exchange chromatographic column (2.6 cm × 20 cm; GE Healthcare, Sweden) eluted with 0, 0.1, 0.3, and 0.5 M NaCl solutions at a flow rate of 2 mL/min. Each 4 mL of the elution was collected and then subjected to carbohydrates content measurement by the phenol–sulfuric acid method. The major peak named EPS-F2 were pooled, dialyzed, and then lyophilized. The EPS-F2 was further purified by gel filtration chromatography using a Sephacryl S-400 HR column (1.6 cm × 60 cm; GE Healthcare, Sweden). The elution was performed using ultrapure water at a flow rate of 1 mL/min (Gan et al., 2020).

### Determination of Basic Components in Exopolysaccharide

The total carbohydrate, protein, and sulfate contents were determined by the phenol-sulfuric acid colorimetric method, Bradford assay, and barium chloride-gelatin colorimetric assay. The UV spectrum of the EPS-F2 solution (5 mg/ml) was measured by a spectrophotometer at a wavelength of 200–800 (U-3900H, Hitachi, Japan).

### Structural Characterization

#### Determination of Homogeneity and Molecular Weight

The homogeneity and average molecular weight of EPS-F2 were determined by high performance size-exclusion chromatography coupled with a differential refractive index and multi-angle laser light scattering detectors. The EPS-F2 sample was dissolved in 0.1 mol/L NaNO_3_ solutions at 25°C and then filtered through a 0.22 μm filter. Subsequently, 100 μL of EPS-F2 solution (5 mg/mL) was loaded onto the Shodex OHpak SB-803 separation system connected to a series of SB-804 and SB-805 separation systems at 60°C. NaNO_3_ solution (0.1 mol/L) was used as the mobile phase; at a flow rate of 0.4 mL/min.

#### Monosaccharide Composition Analysis

Monosaccharide composition of EPS-F2 was determined by high performance anion-exchange chromatography with pulsed amperometric detection. Briefly, the EPS-F2 (5 mg) was hydrolyzed by 1 mL of 2.0 M trifluoroacetic acid and then heated at 121°C for 2 h in a sealed glass ampoule. Excess acid was removed using methanol and then dried using a nitrogen blowing instrument. This process was repeated thrice, and the final hydrolyzed products were dissolved in distilled water. The hydrolyzate and standards were analyzed by a Dionex ICS-5000 ion chromatography system (Thermo Scientific, United States) equipped with a Dionex CarboPac PA-20 analytical column and a Dionex ED50A electrochemical detector.

#### Fourier Transform Infrared Spectroscopy

Fourier transform infrared (FT-IR) spectra of EPS-F2 were recorded on an infrared spectrometer (Nicolet iS1S0, Thermo Nicolet Inc., United States) at the wavenumber, ranging from 500 to 4000 cm^–1^ at room temperature with a total 32 scans (Hao et al., 2020).

#### Glycosyl Linkage Analysis

Glycosyl linkage analysis was performed according to a previously reported method (Hajji et al., 2019), with minor modifications. Briefly, the 10 mg of the sample was vigorously dissolved in 5 mL of anhydrous dimethyl sulfoxide at room temperature. Later, 10 mg of NaOH was added to the mixture and stirred for 1 h. The sample was methylated by adding 3 mL of methyl iodine and maintained at room temperature for 1 h. The reaction product was treated with three volumes of methylene chloride and then dried using a vacuum rotary evaporator. The methylated EPS-F2 sample was further hydrolyzed by 2 M TFA at 120°C for 1.5 h. The products were converted to partially methylated alditol acetates through NaBD4 reduction and acetic anhydride acetylation. The obtained PMAAs were injected into a gas chromatography–mass spectrometer (GC–MS; 6890A-5975C, Agilent Technologies, United States) equipped with an HP-5MS capillary column. The temperature gradients were programmed as follows: 140°C was initially maintained for 2 min and then increased to 230°C at a ramping up rate of 3°C/min.

#### Nuclear Magnetic Resonance

Approximately 30 mg of lyophilized EPS-F2 sample was dissolved in 500 μL of deuterium oxide (D_2_O, 99.9% D) and transferred to an nuclear magnetic resonance (NMR) tube for NMR analysis. 1D NMR (1H- and 13C NMR) and 2 D NMR (COSY, TOCSY, NOESY, HSQC, and HMBC) were determined by a Bruker 800 MHZ spectrometer (Bruker, Switzerland) (Huo et al., 2020).

#### X-Ray Diffraction

X-ray diffraction (XRD) data of EPS-F2 were recorded using an X-Ray diffractometer (D8 ADVANCE Bruker, Germany) equipped with a Cu Ka radiation source (λ = 1.5406 Å). The EPS-F2 was scanned at an angular angle ranging between 5° and 80° at a step size of 0.02°/min.

#### Thermal Properties

Thermogravimetric analysis (TGA) and Differential thermal analysis (DTG) were performed between 50 to 800°C on TG 209F3 Tarsus equipment (NETZSCH, Germany) (Yang et al., 2021). Differential scanning calorimetry (DSC) analysis was conducted using a DSC 214 (NETZSCH, Germany). An EPS-F2 sample was heated at room temperature to 260°C on an alumina pan from at a heating rate of 10°C/min.

#### Zeta Potential, Particle Size and Scanning Electron Microscopy

The zeta potential and particle size of EPS-F2 (0.5%w/v) were determined at 25°C using a NanoPlus Zeta Potential and a particle size analyzer (ZEN5600, Malvern Instruments, United Kingdom), respectively. The surface morphology and microstructure of EPS-F2 (5 mg) were visualized using a scanning electron microscope (SEM, Inspect F50, United States) at an accelerating voltage of 15 kV at 200× and 1000× magnifications (Gunasekaran et al., 2021).

### Determination of Functional Properties

#### Water Solubility Index and Water Holding Capacity

Water solubility index was determined according to a previously reported method (Wang et al., 2010), with minor modifications. An EPS-F2 sample (100 mg) was placed in a centrifuge tube, and 2 mL of deionized water was added into the tube. After the sample was completely dissolved in water, the solution was centrifuged at 12,000 × *g* for 10 min. The supernatant was lyophilized overnight. The WSI was calculated by the following equation:


WSI(%)=(Drysolidweight/Totaldrysampleweight)×100.

According to the method reported by Insulkar et al. (2018), 1.5 mL of ultrapure water was added to 75 mg of EPS-F2 in a centrifuge tube of known weight. The sample was dispersed using a vortex mixer for 5 min. Later, the mixture was centrifuged at 15,000 × *g* for 30 min. Afterward, the supernatant was discarded, and the residues were weighed. The WHC was calculated by the following equation:


WHC(%)=(Waterboundweight/Totaldrysampleweight)×100.

#### Oil Holding Capacity

The OHC of EPS-F2 was determined according to the method reported by Gan et al. (2020), with minor modifications. Briefly, 300 mg of the EPS-F2 sample was placed in a centrifuge tube, and 6 mL of soybean oil was then added to the tube. The sample was mixed using a vortex mixer for 5 min until uniformly dispersed. After centrifugation at 3500 × *g* for 10 min, the supernatant was discarded, and the residue was weighed. The OHC was calculated by the following equation:


OHC(%)=(Oilboundweight/Totaldrysampleweight)×100.

#### Emulsifying Activity

The EA of EPS-F2 was determined according to a previously reported method (Lobo et al., 2019). Briefly, 2 mL of EPS-F2 solution (2 mg/mL) was mixed with various edible oils (soybean oil, palm oil, rap oil, peanut oil, sunflower oil, corn oil, and olive oil) at a ratio of 2:3. The mixtures were vortexed until homogeneous, and their EA was analyzed using the corresponding emulsification index after 1, 24, 48, 72, and 168 h. The EA was calculated by the following equation:


EA(%)=(emulsionlayervolume/totalliquidvolume)×100.

### Rheological Properties

The rheological properties of EPS-F2 samples were determined by an air-bearing MCR 302 stress-controlled rheometer (Anton Paar Germany GmbH, Ostfildern, Germany) equipped with a parallel plate (diameter = 50 mm, gap = 0.05 mm). Additionally, the effects of shear rates (from 0.01 to 200 s^–1^) on the apparent viscosity of EPS at different concentrations (from 0.5 to 4%) were determined. The effects of temperatures (15, 25, 35 and 45°C), pH values (4, 6, 7, 8, and 9), and salts (KCl and CaCl_2_) at different concentrations (0, 0.1, 0.3, and 0.4 M) on the apparent viscosity of 3% EPS-F2 were assessed.

The viscoelastic properties of the EPS-F2 solutions (2.5, 3, and 3.5%) were determined using strain sweep measurements at logarithmically varying shear strains from 0.1 to 100%. The dynamic frequency sweep was performed at angle frequencies ranging from 0.1 to 100 rad/s to obtain the storage moduli (G’) and loss moduli (G”). The effects of KCl (0, 0.3, and 0.4 M) on both G’ and G” of EPS-F2 (3%) were investigated. Subsequently, the temperature ramp oscillation analyses of EPS-F2 solutions (3 and 3.5%) were performed at a fixed strain (1%) and at an increasing temperature from 5 to 80°C.

### Statistical Analysis

All the experiments were performed in triplicate, and the data were expressed as means ± SD. Origin Pro (version 8.5) software (Stat-Ease Inc., Minneapolis, United States) was employed to summarize the data.

## Results and Discussion

### Strain Identification and Exopolysaccharide Production

**FIGURE 1:**
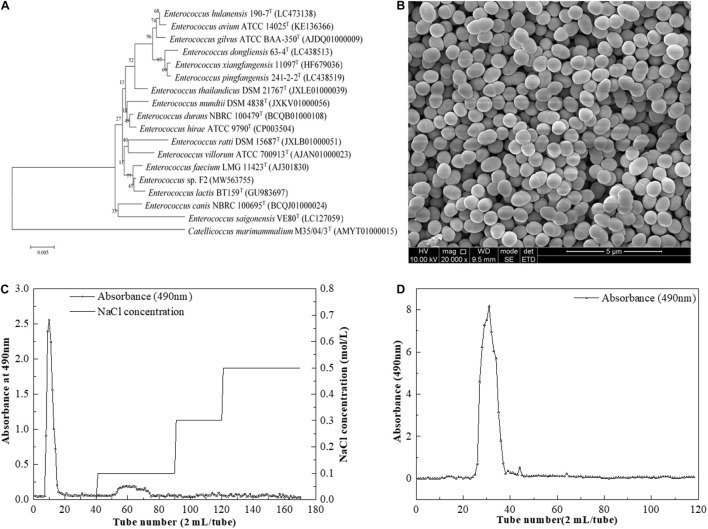
**(A)** Neighbor-joining tree based on 16S rDNA sequences showing genetic relatedness between *Enterococcus* sp. F2 and related species; **(B)** SEM images of *Enterococcus* sp. F2 **(C)** DEAE-52 anion-exchange chromatogram; **(D)** Sephacryl S-400 HR chromatographic profile.

The phylogenetic tree was constructed by the neighbor-joining method based on the 16S rDNA sequences of the strain F2. The phylogenetic tree (Figure 1A) demonstrated that the strain F2 (GenBank accession number MW563755) was closest to *Enterococcus lactis* BT159 (99.65% 16S rRNA gene). Thus, it was named *Enterococcus* sp. F2. This strain was observed in either pairs or short chains (Figure 1B).

**FIGURE 2:**
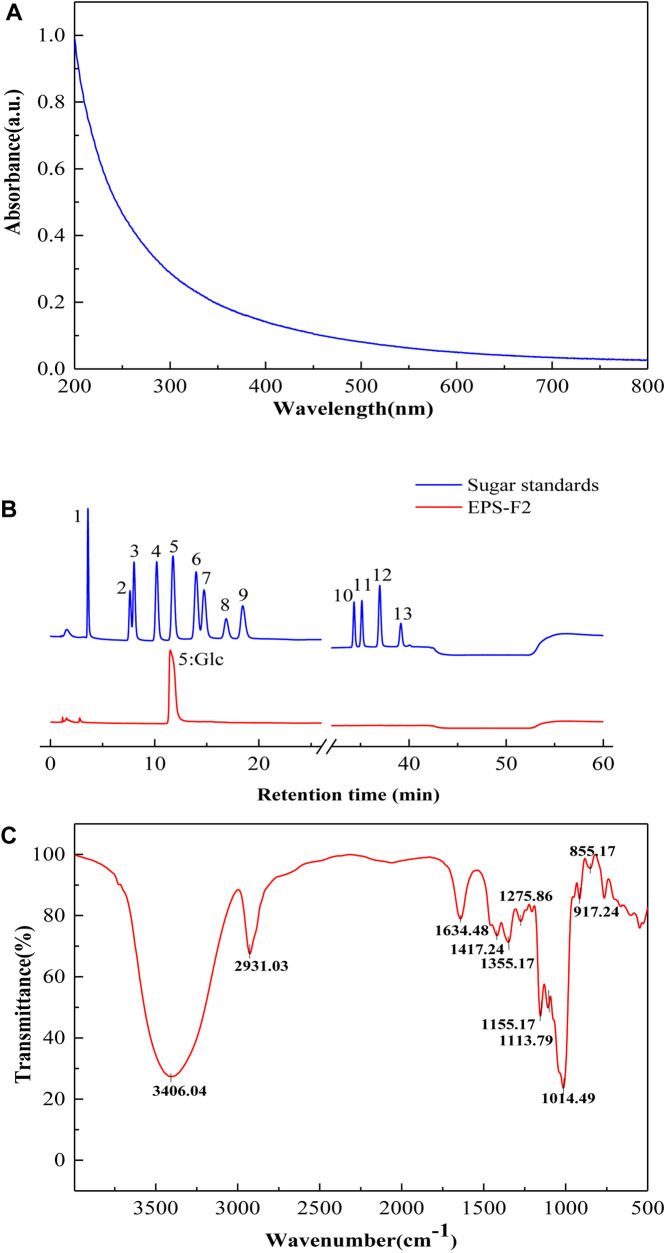
**(A)** UV – vis absorption spectrum; **(B)** HPAEC-PAD profiles of monosaccharide standards and EPS-F2 (Peak identities:1, Fucose; 2, Rhamnose; 3, Arabinose; 4, Galactose; 5, Glucose; 6, Xylose; 7, Mannose; 8, Fructose; 9, Ribose; 10, Galacturonic acid; 11, Guluronic Acid; 12, Glucuronic acid; 13, Mannuronic acid.) **(C)** FTIR spectrum of EPS-F2 sample.

After pretreatment, the sample was first separated through the DEAE-Sepharose Fast Flow ion-exchange column (Figure 1C), and the major peak fractions were pooled and further purified by a Sephacryl S-400 HR gel-filtration column (Figure 1D). The elution profile appeared as one symmetrical peak, indicating that the sample was homogeneous. The basic component analysis results showed that the total sugar content of EPS-F2 was 97.71 ± 1.1%, while sulfates and proteins were not detected in the purified EPS-F2. The UV spectrum of EPS-F2 solution (Figure 2A) did not show any absorption peaks at 260 nm and 280 nm, indicating the absence of nucleic acids and proteins or a high pure sample.

### Molecular Weight and Monosaccharide Composition

As shown in Supplementary Figure 1, the single and symmetrical peak indicated that the purified EPS-F2 was highly homogeneous. The polydispersity index (Mw/Mn) was close to 1 (1.017; Table 1), verifying its narrow molar mass distribution. The weight-average molecular weight (Mw) of EPS-F2 was 1.108 × 10^8^ g/mol (Table 1), which was larger than the exopolysaccharide from *E. faecium* MS79 (8.3 × 10^5^ g/mol) (Ayyash et al., 2020c) and *E. faecalis* DU10 (2.26 × 10^5^ g/mol) (Venkatesh et al., 2016). According to a previous report, EPS with a larger molecular weight plays an essential role in the food industry due to their distinctive rheological properties and viscosity (Zhou et al., 2019). In consistent with this result, the EPS-F2 isolated in this study had a relatively high Mw. Therefore, it was inferred that it might exhibit excellent rheological properties.

**TABLE 1:** Relevant molecular parameters of the purified EPS-F2 in HPSEC-RI-MALLS analysis.

**Characteristic**	**Parameter**	**Detection results**
Molar mass moments (g/mol)	Mw	1.108 × 10^8^
	Mn	1.089 × 10^8^
	Mz	1.125 × 10^8^
	Mp	1.178 × 10^8^
Polydispersity index	Mw/Mn	1.017
	Mz/Mn	1.032
RMS radius moments (nm)	Rw	81.3
	Rn	81.7
	Rz	80.9

As depicted in Figure 2B, HPAEC-PAD analysis showed that the only one sugar in EPS-F2 was glucose, manifesting that it was glucan. The monosaccharide composition of EPS-F2 differed from *E. faecium* MS79 (Ayyash et al., 2020c) and *E. faecalis* EJRM152 (Kansandee et al., 2019). These results demonstrated that the compositions of EPS monosaccharides produced by LAB are affected by species, media ingredients, and culture conditions.

### Fourier Transform Infrared Spectrum and Glycosyl Linkage of Exopolysaccharide-F2

As illustrated in the FT-IR spectra (Figure 2C), EPS-F2 exhibited a broad and intense peak at 3406.04 cm^–1^ due to the stretching vibration of O–H in its EPS chain (Vettori et al., 2012). A strong absorption band at 2931.03 and 1417.24 cm^–1^ could be ascribed to the C–H stretching vibration, and the band in the 1634.48 cm^–1^ regions could be ascribed to the associated water (Chen et al., 2021). The structural characterization of polysaccharides was determined by two major bands at 1200–950 and 950–750 cm^–1^. The absorption band observed at 1155.17 cm^–1^ might be attributed to the stretching and bending vibration of the C–O–C bond and glycosidic bridge (Gu et al., 2020). A weak characteristic peak at 1113.79 cm^–1^ might be attributed to the presence of the C–O bond in the glucose residue (Tian et al., 2016). A strong absorption peak at 1014.49 cm^–1^ indicated that this dextran (EPS-F2) contained α-(1→6) glycosidic bonds (Miao et al., 2014). Additionally, the absorption peak at 855.17 and 917.24 cm^–1^ indicated the presence of the α-configuration of pyranose glycosidic residues (Chen et al., 2013). Overall, these results suggested that EPS-F2 contained α-(1→6) linkages; however, this was further confirmed by the glycosidic bond type analysis and NMR.

The types of glycosidic bonds in EPS-F2 derived from the methylation analysis are summarized in Table 2 and Supplementary Figure 2. These results suggested that EPS-F2 had three major derivatives, namely 1,5-di-*O*-acetyl-2,3,4,6-tetra-*O*-methyl glucitol, 1,5,6-tri-*O*-acetyl-2,3,4-tri-*O*-methyl glucitol, and 1,3,5,6-tetra-*O*-acetyl-2,4-di-*O*-methyl glucitol at a molar ratio of 10.45:84.82:4.73. Based on these results, it was concluded that the major bond types in EPS-F2 consisted of T-Glc*p*-(1→ (10.45%), →6)-Glc*p*-(1→ (84.82%), and →3,6)-Glc*p*-(1→ (4.73%) and that the main chain of EPS-F2 consisted of glucopyranose units linked by α-(1–6) linkages and partially branched at the *O*-3 position with a single α-glucopyranose unit.

**TABLE 2:** Glycosidic linkage composition of methylated EPS-F2 by GC–MS analysis.

**Time (min)**	**Methylated sugars**	**Deduced linkages**	**Molar ratios**	**Major mass fragments (*m/z*)**
9.48	2,3,4,6-Me_4_-Glc*p*	T-Glc*p*-(1→	10.45	87, 102, 129, 145, 162, 205
14.54	2,3,4-Me_3_-Glc*p*	→6)-Glc*p*-(1→	84.82	71, 87, 102, 118, 162, 189, 203, 233
18.47	2,4-Me_2_-Glc*p*	→3,6)-Glc*p*-(1→	4.73	74, 87, 101, 118, 160, 189, 202, 234, 305

### Nuclear Magnetic Resonance Spectroscopic Analysis of Exopolysaccharide-F2

**FIGURE 3:**
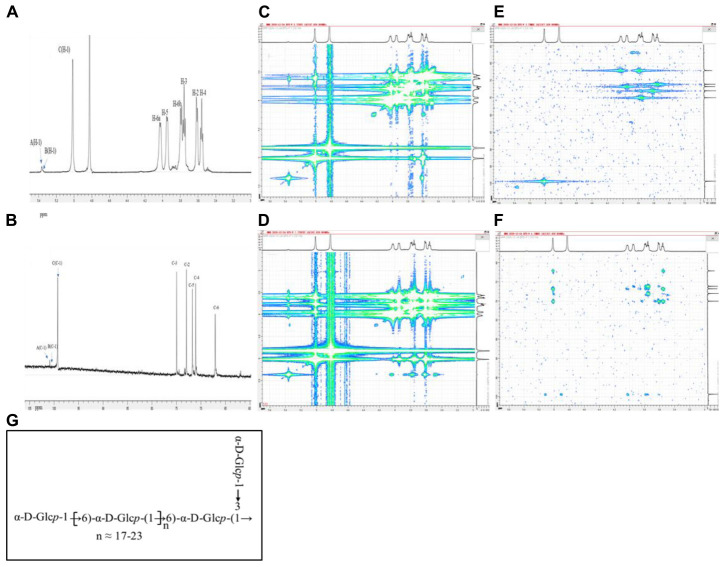
NMR spectra of EPS-F2 recorded in D_2_O at 298 K: **(A)** 1D-^1^H spectrum and; **(B)** 1D-^13^C spectrum; **(C)** COSY spectrum; **(D)** TOCSY spectrum; **(E)** HSQC spectrum; **(F)** HMBC spectrum; **(G)** one putative structure of EPS-F2.

The structural characteristics of EPS-F2 were further elucidated by various 1D and 2D NMR techniques based on the methylation analysis results. According to the ^1^H NMR (Figure 3A) and ^13^C NMR (Figure 3B) spectra, the chemical shifts (δ) for anomeric proton (H-1) and carbon (C-1) were observed between δ 4.97–5.39 and δ 99.26–101.83 ppm, respectively, while those for H-2 to H-6 and C-2 to C-6 protons, these shifts were observed between δ 3.45–4.04 and δ 61.86–81.50 ppm, respectively. The signals of the three anomeric protons were observed at δ 5.37, δ 5.35, and δ 5.01 ppm, labeled as A, B, and C, respectively. Other proton signals were confirmed by COSY (Figure 3C) and TOCSY (Figure 3D), while the carbon signals associated with hydrogen signals were confirmed by the HSQC experiment (Figure 3E).

The chemical shift of anomeric C residue at δ 5.01 ppm and its related signal at δ 99.36 ppm was observed in the HSQC spectrum, indicating that the sample has α-configuration. The associated signals at δ 5.01/3.62, δ 3.62/3.76, δ3.76/3.56, δ 3.56/3.96, δ3.96/4.02 (3.79) ppm of residue C were obtained from the ^1^H-^1^H COSY spectrum by combining the TOCSY spectral data. Based on these results, the signals at δ 3.62, δ 3.76, δ 3.56, δ 3.96, and δ 4.02 (3.79) ppm were assigned to H–2, H–3, H–4, H–5, and H–6 of residue C, respectively. The corresponding chemical shifts of C–2, C–3, C–4, C–5, and C–6 were δ 73.05, δ 74.96, δ 71.13, δ 71.83, and δ 67.04 ppm, respectively. Compared with the previously reported data of glucose units (Yang et al., 2015; Wang et al., 2017), the signal of C-6 (δ 67.04 ppm) shifted downward, suggesting that residue C was replaced at the C-6 position. Thus, it was deduced as →6)-α-D-Glc*p*-(1→.

As for residue B, the chemical shifts of H-1 to H-6 were assigned to δ 5.35, δ 3.60, δ 3.88, δ 3.49, δ 3.94, and δ 4.00 (3.81) ppm, respectively by combining the results in the TOCSY and COSY spectra. Based on these chemical shifts, the ^13^C chemical shifts obtained by HSQC spectrum were assigned to δ 100.51, δ 72.50, δ 82.18, δ 71.10, δ 71.74, and δ 66.80 ppm, respectively. The relative downward shifts of C-3 (δ 82.18 ppm) and C-6 (δ 66.80 ppm) indicated that residue B was →3, 6)-α-D-Glc*p*-(1→ (C. Zhao et al., 2006; B. Wang et al., 2019; Liu et al., 2021). Moreover, the signal of the linkage in residue A was verified by similar methods, and all data are summarized in Table 3.

**TABLE 3:** ^1^H and ^13^C NMR chemical shift data for EPS-F2.

**Sugar residue**	**Chemical shifts δ (ppm)**						
	**H-1/C-1**	**H-2/C-2**	**H-3/C-3**	**H-4/C-4**	**H-5/C-5**	**H-6/C-6**	**H-6′/C-6**
A: -α-D-Glc*p*–(1→	5.37/100.88	3.60/73.50	3.74/74.73	3.46/71.07	3.86/71.01	3.87/61.96	3.78
B: →3,6)-α-D-Glc*p*-(1→	5.35/100.51	3.60/72.50	3.88/82.18	3.49/71.10	3.94/71.74	4.00/66.80	3.81
C: →6)-α-D-Glc*p*-(1→	5.01/99.36	3.62/73.05	3.76/74.96	3.56/71.13	3.96/71.83	4.02/67.04	3.79

The overlapping of the carbon and proton signals within the sugar residues was observed in the HMBC spectrum (Figure 3F), and the linkages (as well as their sequences) among different residues were elucidated. The cross-peaks between H-1 (δ 5.01 ppm) and C–6 (δ 67.04 ppm) of residue C, between H-3 (δ 3.88 ppm) of residue B and C–1 (δ 100.88 ppm) of residue A, and between H–1 (5.37 ppm) of residue A and C–3 (δ 82.18 ppm) of residue B indicated the presence of C-(1→6)-C and A-(1→3)-B linkages. Furthermore, the cross-peaks between H–1 (δ 5.35 ppm) of residue B and C–6 (δ 67.04 ppm) of residue C, and between H-1 (δ 5.01 ppm) of residue C and C–6 (δ 66.80 ppm) of residue B indicated the presence of C–(1→6)–B–(1→6)–C linkages in the back-bone of this exopolysaccharide. Based on the methylation and NMR spectra analyses results, a putative structure of EPS-F2 was proposed, as illustrated in Figure 3G.

### Thermal Stability of Exopolysaccharide-F2

**FIGURE 4:**
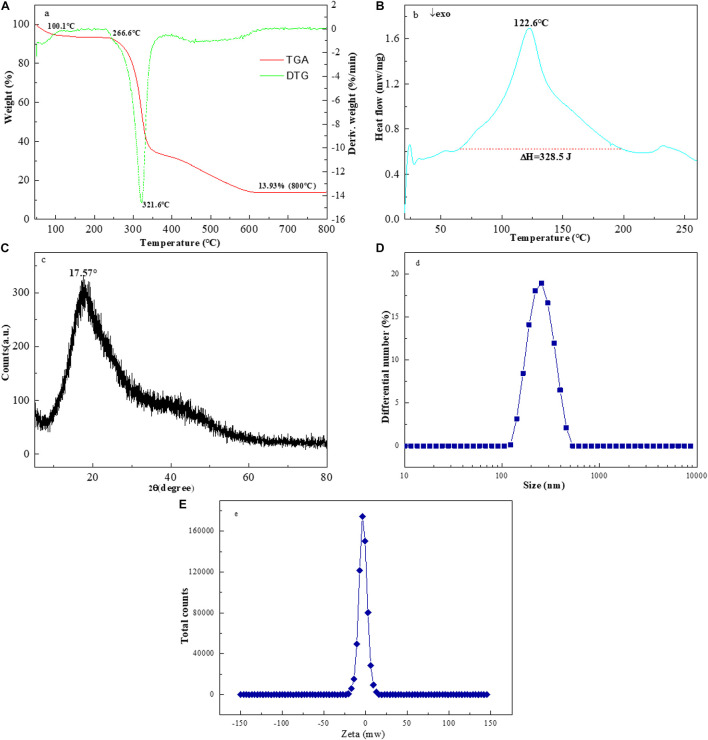
The TGA **(A)**, **(B)** DSC and **(C)** XRD spectra of EPS-F2, **(D)** Particle size distribution and **(E)** zeta potential trend of EPS-F2 in ultrapure water.

As illustrated in Figure 4A, an approximate weight loss of 5.4% occurred between 50.0 and 100.1°C temperatures, which might be related to the loss of adsorbed and bound water in the EPS-F2 sample. This result indicated that the sample was rich in carboxyl groups, thus increasing its WHC. Nevertheless, a significant weight change of EPS-F2 was not observed at 100.1 and 266.6°C temperatures, suggesting that the sample remained relatively stable at temperatures below 266.6°C. This result demonstrated that EPS-F2 should not be used at temperatures near or above 266.6°C to maintain the complete structure of dextran. The maximum weight loss of about 75.75% was observed when the temperature was raised from 266.6 to 600°C. This was likely because of the depolymerization of the EPS-F2 sample, followed by the breakdown of chemical bonds in the sugar ring (Miao et al., 2015). Based on the DTG curve, a sharp peak at 321.6°C indicated that the degradation temperature (Td) of EPS-F2 was 321.6°C, which was higher than dextran from *Weissella cibaria* MED17 (300°C) (Aburas et al., 2020) and *Lactobacillus reuteri* SK24.003 (292.6°C) (Miao et al., 2015). This difference could possibly be due to the variance in molecular configurations of the polymer. This phenomenon is vital for the food industry as a higher Td could make the EPS-F2 potentially be suitable for a number of applications, such as edible film materials, coating packaging materials, and food additives.

Figure 4B illustrates the DSC thermograms of EPS-F2 between 25 to 260°C temperatures. A significant endothermic peak was observed at 122.6°C, and an endothermic enthalpy change (ΔH), the amount of heat required to melt 1 g of EPS-F2, was found to be 328.5 J. Mannan from *Weissella confusa* MD1 has a melting point of 267.64°C and a melting enthalpy change (ΔH) of 337.7 J/g (Lakra et al., 2020). Kanmani et al. (2011) have described that the melting temperature of EPS from *Streptococcus phocae* PI80 (arabinose, fructose, and galactose) is 120.09°C, and its ΔH is 404.6 J/g. These differences in the melting temperatures and ΔH values might be attributed to the different monosaccharide compositions. Zhou et al. (2018) have revealed that the endothermic peak and enthalpy change of *Leuconostoc pseudomesenteroides* XG5 dextran were 274.14°C and 101.80 J/g, respectively. This finding strongly supports the view that the thermal behavior of EPS from various lactic acid bacteria strains might be different.

### X-Ray Diffraction, Particle Size, Zeta Potential and Scanning Electron Microscopy Analysis of Exopolysaccharide-F2

**FIGURE 5:**
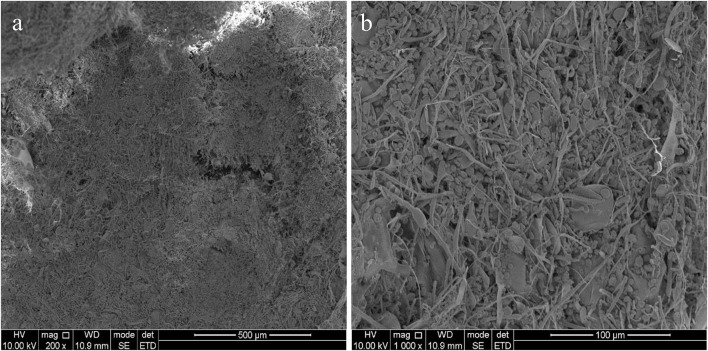
SEM images of EPS-F2 under 200× **(a)** and 1000× **(b)** magnification.

The X-ray diffractograms of EPS-F2 are presented in Figure 4C. The EPS-F2 sample exhibited a relatively broad peak at 2θ = 17.57°, indicating that it had an amorphous/crystalline structure. The size distribution analysis suggested that the average particle diameter of EPS-F2 was 235 ± 3.6 nm (Figure 4D), which was lower than the EPS from *Bacillus paralicheniformis* SR14 (293.73 ± 4.0 nm) (Cheng et al., 2017) and larger than the EPS from *Lactococcus garvieae* C47 (166.6 nm) (Ayyash et al., 2020a). This result suggests that size distribution could depend on molecular weights, types of glycosyl linkages, and monosaccharide compositions. As illustrated in Figure 4E, EPS-F2 had a negative zeta potential value of −3.44 mW. The negative charge of EPS-F2 was most likely caused by the functional groups in its sugar ring and its functional properties. The surface morphology of EPS-F2 was observed under 200× and 1000× magnifications, as presented in Figure 5. EPS-F2 had a relatively rough, porous, and branched morphology, indicating its excellent physicochemical properties, such as WHC, OHC, and viscosity. In contrast, Ye et al. (2018) have reported that dextran has a porous and smooth surface. The difference might be due to the use of different procedures in handling the sample, especially for the sample extraction or purification.

### Functional Properties of Exopolysaccharide-F2

The WSI of EPS-F2 was 14.62 ± 0.33% (Supplementary Table 1), which was much lower than the dextran produced by *Weissella cibaria* YB-1 (95.23 ± 4.45%) (Ye et al., 2018), and higher than the dextran produced by *Lactobacillus kefiranofaciens* ZW3 (14.2%) isolated from Tibet kefir (Ahmed et al., 2013). Previous studies have reported that several factors lead to the differences in WSI of EPS, such as chain lengths, glycosidic linkages arrangement, and the secondary and tertiary structures. The WHC of EPS-F2 was 882.5 ± 20.44%, which was higher than the dextran from *Leuconostoc citreum* B-2 (450%) (Feng et al., 2018) and *Leuconostoc lactis* L2 (509.45 ± 28.59%) (Zhao et al., 2019). Therefore, it was inferred that the relatively high molecular weight of EPS-F2 might attribute to its excellent WHC. The OHC of EPS-F2 was 1867.76 ± 11.33%, which is the highest OHC of EPS from lactic acid bacteria. Factors leading to the difference in OHC include the porosity of fiber structure and the chemical composition of biopolymer (Jeddou et al., 2016). Therefore, EPS-F2 in fat-containing food should be widely exploited to maintain and enhance its flavors, extend its shelf life, and improve its mouthfeels.

Exopolysaccharide have the advantages of significant bioemulsifiers for different industrial applications due to their emulsion stabilization ability between water and hydrophobic compounds. In this study, the emulsification activity of EPS-F2 determined against soybean oil, palm oil, rap oil, peanut oil, sunflower oil, corn oil, and olive oil (Table 4) for 1 h were 85.8 ± 1.1, 100.0 ± 0.0, 74.9 ± 0.7, 65.5 ± 0.4, 64.6 ± 0.8, 72.0 ± 0.3, and 92.5 ± 0.4%, respectively. Although its emulsification activity slightly decreased when the sample was stored for 24 h, it is noteworthy that an emulsifier maintaining its capacity to at least 50% of the primal volume of an emulsion after 24 h of its formation is an acceptable emulsifier. The results also showed that EPS-F2 had a high emulsifying ability in all tested oils, especially in soybean oil, palm oil, and olive oil. It should be noted that no studies have been reported so far on the emulsification activity of dextran from *Enterococcus* species. However, there is evidence of reporting dextran produced by other bacterial strains. For instance, dextran produced by *Weissella cibaria* YB-1 has high emulsification activity in sunflower oil (83.43 ± 3.65%) (Ye et al., 2018). Therefore, it was speculated that EPS produced by *Enterococcus* sp. F2 might have potential applications in diverse industrial sectors as an emulsifier in the future.

**TABLE 4:** Emulsifying capacity of EPS-F2 and its stability against tested oils.

**Oils**	**Emulsification activity**				
	**EA_1_**	**EA_24_**	**EA_48_**	**EA_72_**	**EA_168_**
Soybean oil	85.8 ± 1.1^c^	75.4 ± 0.3^c^	69.7 ± 0.6^c^	61.3 ± 1.3^c^	60.7 ± 1.8^b^
Palm oil	100.0 ± 0.0^a^	91.5 ± 0.2^a^	83.9 ± 0.4^a^	83.2 ± 0.8^a^	77.6 ± 0.6^a^
Rap oil	74.9 ± 0.7^d^	69.8 ± 1.1^d^	63.7 ± 0.7^d^	58.1 ± 0.7^d^	54.1 ± 0.2^c^
Peanut oil	65.5 ± 0.4^f^	58.0 ± 0.3^e^	54.4 ± 1.1^e^	51.3 ± 1.3^e^	44.5 ± 2.5^d^
Sunflower oil	64.6 ± 0.8^f^	54.4 ± 1.2^f^	46.0 ± 0.6^f^	42.3 ± 0.1^f^	37.9 ± 0.4^e^
Corn oil	72.0 ± 0.3^e^	68.8 ± 1.9^d^	63.7 ± 0.4^d^	62.5 ± 0.7^c^	56.9 ± 1.5^c^
Olive oil	92.5 ± 0.4^b^	77.7 ± 0.8^b^	70.5 ± 2.1^b^	70.0 ± 2.0^b^	62.5 ± 1.1^b^

*^*a*−*f*^Means ± SD within a column with different lowercase superscript differ (*P* < 0.05).*

### Steady Shear Flow Behavior of Exopolysaccharide-F2

Generally, the applicability of EPS as a gelling agent, stabilizer, and thickener in food products and film-forming substrates is dependent on their rheological properties in an aqueous solution. Therefore, determining the rheological properties of EPS has significant importance.

**FIGURE 6:**
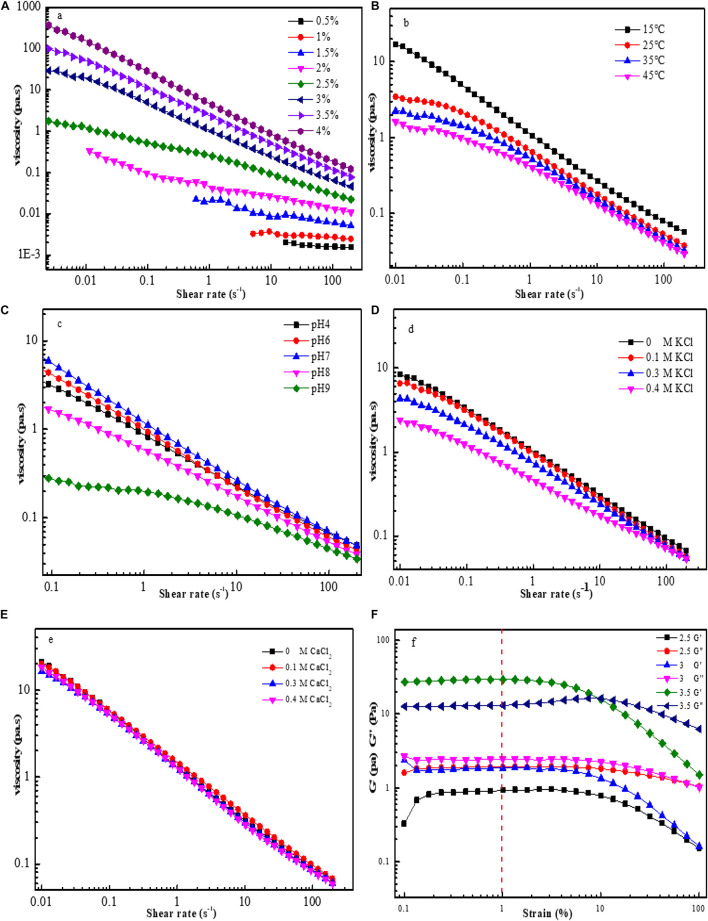
Flow curves for EPS-F2 samples at various conditions: Effect of concentrations on apparent viscosity of EPS-F2 **(A)**; Effect of temperature on apparent viscosity of EPS-F2 **(B)**; Effect of pH on apparent viscosity of EPS-F2 **(C)**; Effect of K^+^ on apparent viscosity of EPS-F2 **(D)**; Effect of Ca^2+^ on apparent viscosity of EPS-F2 **(E)**; The linear viscoelastic region curves of EPS-F2 **(F)**.

The rheological properties of EPS-F2 at different concentrations (0.5–4%) were determined at 25°C in terms of the steady flow behavior. As illustrated in Figure 6A, the apparent viscosity of the samples increased with the increase of concentration, while decreased with the increase of shear rate (up to 200 s^–1^). This result indicates that EPS-F2 has typical shear thinning or non-Newtonian fluid behavior, which is the typical behavior of EPS in aqueous solutions due to the orientations of their molecular components. Moreover, increasing the shear rate could alter the entanglement network structure between the molecular chains, leading to the random orientation of the molecular chains and decreased viscosity due to the decrease in the interaction of adjacent chains (Wang et al., 2020). Pseudoplasticity can be fitted to the power-law model: τ = k(γ)^n^; where τ, γ, and K are the shear stress (mPa), the shear rate (s^–1^), and the consistency index parameter (mPa S^n^), respectively. The flow behavior index (n) is the degree of non-Newtonian behavior: when 0 < n < 1, the fluid is non-Newtonian pseudoplastic. As summarized in Supplementary Table 2, EPS-F2 had 0 < n < 1, indicating its non-Newtonian property.

The results of 3% EPS-F2 aqueous solutions exposed to 15, 25, 35, and 45°C temperatures are illustrated in Figure 6B, which revealed that the apparent viscosity decreased with the increase of temperature. This might be attributed to the fact that an increase in the temperature increases the polysaccharide chain extension, thereby increasing the volume of the molecule; as a result, the intermolecular interaction forces and the apparent viscosity are reduced (Ji et al., 2019). It is noteworthy that the sample could still maintain its non-Newtonian fluid behavior, despite the increase in temperature. Furthermore, the consistency index parameter (K) decreased, and the flow behavior index (n) increased with the increase in temperature (Supplementary Table 2). These findings suggest that an increase in temperature can reduce the thickening and pseudoplastic properties of EPS-F2.

As depicted in Figure 6C, the apparent viscosity obtained at neutral condition (pH 7) was higher than that obtained at acidic (pH 4 and 6) or alkaline (pH 8 and 9) conditions. At neutral conditions, the electrostatic repulsion between the anionic groups in EPS could lead to higher viscosity; but at acidic or alkaline conditions, the protonation of charged groups in EPS is weakened, thus allowing the EPS chain to have more flexibility and reducing the apparent viscosity (Abid et al., 2021). Irrespective of these phenomena, the appropriate apparent viscosity of the sample and its pseudoplasticity were maintained (Supplementary Table 2). Similarly, Li et al. (2017) have reported that EPS from *Paenibacillus edaphicus* NUST16 has higher viscosity in near neutral conditions.

The effects of K^+^ at different concentrations on the viscosity of EPS-F2 are illustrated in Figure 6D. According to the figure, the apparent viscosity of EPS-F2 decreased upon the addition of K^+^ at low shear rates, and the decrease became more prominent at higher K^+^ concentrations (0.3 and 0.4 M). It is apparent that the addition of K^+^ could weaken the electrostatic repulsion between molecules, causing them to expand and reducing the viscosity (Wang et al., 2020). At high shear rates, the apparent viscosity slightly decreased with increasing K^+^ concentration. Therefore, the apparent viscosity of EPS-F2 was affected by K^+^ concentrations and shear rates. In contrast, the addition of Ca^2+^ at different concentrations significantly affected the apparent viscosity (Figure 6E). These results indicate that EPS-F2 is more suitable for systems containing divalent salts.

Different EPS-F2 solutions (2.5–3.5%) were subjected to different shear strain rates ranging from 0.1 to 100% (Figure 6F). At the shear strain rate of 1%, the storage modulus (G’) and the loss modulus (G”) remained almost constant, indicating a stable gel network of EPS-F2. Hence, a fixed shear strain rate of 1% was employed in the dynamic oscillation test.

### Dynamic Rheological Behavior Analysis

**FIGURE 7:**
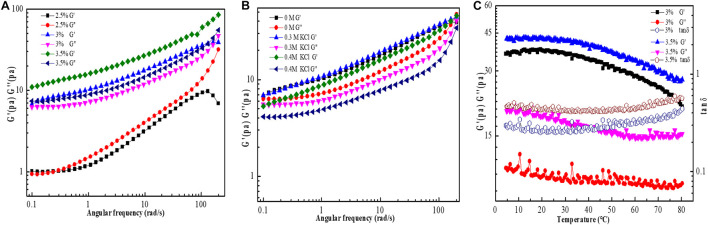
Dynamic frequency sweep of EPS-3 solutions at different concentrations **(A)**; Influence of K^+^ on G’ (storage modulus) and G” (loss modulus) of 3% EPS-F2 solution **(B)**; Temperature dependence of G’ and G” of 3% EPS-F2 solution **(C)**.

The dynamic frequency sweeps results are illustrated in Figure 7A. At a low concentration (2.5%), the G” value was higher than the G’ value over the whole angle frequency range tested (0.1–200 rad/s); reflecting the liquid-like behavior of the sample. In contrast, at higher concentrations (3 and 3.5%), the G’ values were higher than the G” values over the same angle frequency range, indicating the presence of a gel-like structure in the solution system (solid-like behavior) and that the G’ and G” values are angle frequency-dependent. Compared with 3% EPS-F2 solution, 3.5% EPS-F2 solution had higher G’ and G” values and weaker frequency dependency due to stronger hydrogen bonds and electrostatic interactions between molecules at a higher concentration, which in turn strengthen the stability of the gel system (Abid et al., 2021). Similar results were reported by Maalej et al. (2016), suggesting that the gel-like behavior of EPS22 is dependent on the solution concentration.

The results of the effect of K^+^ on the viscoelastic properties of EPS-F2 at 25°C are illustrated in Figure 7B. With the increase of K^+^ concentration, the G’ value of EPS-F2 remained higher than the G” value over the frequency range tested, indicating that EPS-F2 predominantly has elastic behavior. Nevertheless, it was also found that the addition of K^+^ led to a slight decrease in G’ and G” values, which was consistent with the steady rheological properties test results. Li and Hou (2011) have found that the presence of Na^+^ at low concentrations results in a slight increase in the G’ value without affecting the G” value; however, the presence of Na^+^ at high concentrations led to a slight decrease in both values. The difference might be attributed to the different cationic interactions in EPS.

The effects of high temperature on the viscoelastic properties of EPS-F2 at two concentrations (3 and 3.5%) were determined by temperature ramp oscillation. As illustrated in Figure 7C, both G’ and G” values gradually decreased when the temperature was increased from 5°C to 80°C. This result was consistent with the trend of apparent viscosity changes in EPS with temperatures. Additionally, the G’ value was slightly higher than the G” value at the temperature range studied, which suggests that EPS-F2 solution (3 and 3.5%) could maintain its solid-like behavior (gel-like structure) at high temperatures. The viscoelastic behavior was determined by the tan δ (G”/G’) value, directly linked to the system energy loss: tan δ < 1 indicates predominant solid-like behavior, whereas tan δ > 1 indicates predominant liquid-like behavior (Abid et al., 2021). The tan δ values of EPS-F2 solutions (3 and 3.5%) were lower than 1 (Figure 7C), confirming that the sample had a solid-like behavior. The G’ value was higher than the G” value at the plateau over a certain temperature range. However, the G” value was higher than the G’ value when the temperature was significantly increased above 60°C, e.g., 70°C, indicating that the sample had a liquid-like behavior. These behaviors could be explained by the initial swelling of EPS22 solutions, followed by the breakage of some inter- or intra-molecular hydrogen bonds.

## Conclusion

In conclusion, a novel EPS-producing strain F2 was isolated from Guizhou fermented soya beans and identified as *Enterococcus*. The functional properties and rheological properties of EPS-F2 were investigated after purification and characterization. The findings indicated that EPS-F2 consisted of only glucose with an average molecular weight of 1.108 × 10^8^ g/mol. The FTIR, GC–MS, and NMR results confirmed that EPS-F2 contained →6)-α-D-Glc*p*-(1→ linkage in the main chain and →3, 6)-α-D-Glc*p*-(1→ linkage in the branch chain. The thermal properties determination, XRD, and SEM results revealed that it was an amorphous-crystalline biopolymer with excellent thermal stability. Additionally, EPS-F2 possessed significant functional properties, especially for WHC, OHC, and EA. Besides, the steady shear and dynamic oscillatory measurement results revealed the non-Newtonian behavior and liquid-like behavior of EPS-F2, respectively. However, further studies on this glucan are highly warranted to better understand its synthesis mechanism, and structural–functional relationship, and exploration of its potential applications in foods, pharmaceuticals, and other products.

## Data Availability Statement

The original contributions presented in the study are included in the article/Supplementary Material, further inquiries can be directed to the corresponding author/s.

## Author Contributions

GJ contributed to conceptualization, investigation, and writing – original draft preparation. LG contributed to methodology and validation. XL contributed to data curation. JH contributed to software. SZ and JC contributed to investigation and software. RZ and ZX contributed to formal analysis and software. YT contributed to conceptualization, funding acquisition, supervision, writing – reviewing and editing, and project administration. All authors contributed to the article and approved the submitted version.

## Conflict of Interest

The authors declare that the research was conducted in the absence of any commercial or financial relationships that could be construed as a potential conflict of interest.

## Publisher’s Note

All claims expressed in this article are solely those of the authors and do not necessarily represent those of their affiliated organizations, or those of the publisher, the editors and the reviewers. Any product that may be evaluated in this article, or claim that may be made by its manufacturer, is not guaranteed or endorsed by the publisher.
