# Supplementary material for: Characterization of Structural and Physicochemical Properties of an Exopolysaccharide Produced by Enterococcus sp. F2 From Fermented Soya Beans
Source: Front Microbiol. 2021 Oct 29;12:744007. doi: 10.3389/fmicb.2021.744007 (PMC8586432; doi:10.3389/fmicb.2021.744007)
Supplement: Supplementary file 1 [file Data_Sheet_1.docx]

**Supplementary Material for**

**Structural characterization and physicochemical properties of an exopolysaccharide produced by *Enterococcus* sp. F2 from fermented soya beans**

Guangyang Jiang ^a,b^, Longzhan Gan,^a,b^, Xiaoguang Li ^a,b^, Juan He ^c^, Shihao Zhang ^a,b^, Jia Chen ^a,b^, Ruoshi Zhang ^a,b^, Zhe Xu ^a,b^, Yongqiang Tian ^a,b,⁎^

^a^ College of Biomass Science and Engineering, Sichuan University, Chengdu 610065, PR China

^b^ Key Laboratory of Leather Chemistry and Engineering (Sichuan University), Ministry of Education, Chengdu 610065, PR China

^c^ Key Laboratory of Bio-Resources and Eco-Environment of Ministry of Education, College of Life Sciences, Sichuan University, Chengdu, 610065, PR China

**^⁎^Corresponding author: Yongqiang Tian**

**E-mail:** yqtian@scu.edu.cn.

**Address:** No.24 south section 1, Yihuan Road, Chengdu, China

**Fig. S1** Chromatogram of the molar mass distribution of EPS-F2 on HPSEC RI MALLS

system with 0.1 M NaNO_3_ solution as mobile phase at a flow rate of 0. 4 mL/min.

**Fig. S2** Mass spectra for methylation analysis of EPS-F2. (A) T-Glc*p*-(1→; (B) →6)-Glc*p*-(1→;

(C) →3,6)-Glc*p*-(1→


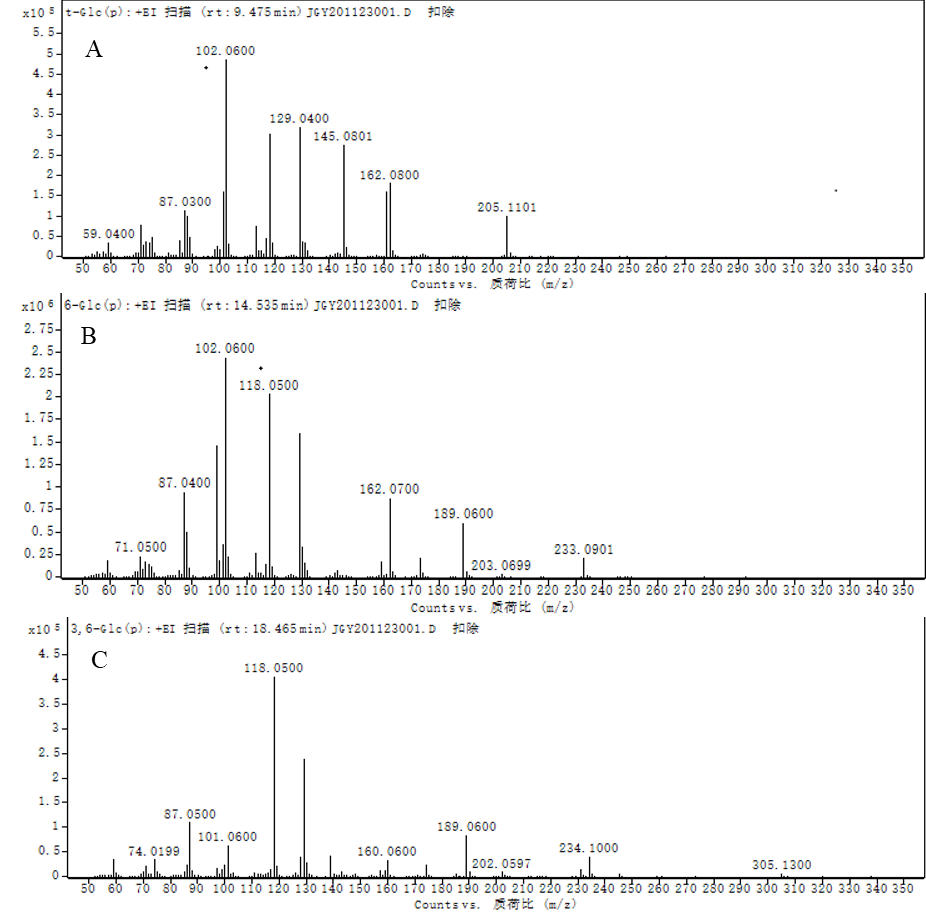


**Table. S1** physicochemical properties of EPS-F2

| sample | WSI (%) | WHC (%) | OHC (%) |
| --- | --- | --- | --- |
| EPS-F2 | 14.62±0.33 | 882.5±20.44 | 1867.76±11.33% |

**Table. S2** The consistency index parameter (K)and flow behavior index (n)) of EPS-F2 solutions under various conditions.

| Variables |  | K | n | R^2^ |
| --- | --- | --- | --- | --- |
| Concentration/% | 0.5 | 0.002±0.001 | 0.936±0.013 | 0.999 |
|  | 1 | 0.004±0.001 | 0.896±0.009 | 0.999 |
|  | 1.5 | 0.017±0.001 | 0.773±0.007 | 0.999 |
|  | 2 | 0.052±0.001 | 0.706±0.002 | 0.999 |
|  | 2.5 | 0.256±0.004 | 0.539±0.003 | 0.999 |
|  | 3 | 0.976±0.019 | 0.418±0.004 | 0.998 |
|  | 3.5 | 2.211±0.033 | 0.361±0.004 | 0.998 |
|  | 4 | 4.491±0.107 | 0.309±0.006 | 0.993 |
| pH | 4 | 0.753±0.016 | 0.479±0.005 | 0.998 |
|  | 6 | 0.845±0.021 | 0.432±0.006 | 0.997 |
|  | 7 | 1.048±0.028 | 0.413±0.006 | 0.996 |
|  | 8 | 0.553±0.006 | 0.495±0.002 | 0.999 |
|  | 9 | 0.253±0.004 | 0.6246±0.003 | 0.999 |
| Temperature/℃ | 15 | 0.935±0.028 | 0.4643±0.007 | 0.996 |
|  | 25 | 0.593±0.011 | 0.475±0.004 | 0.998 |
|  | 35 | 0.496±0.007 | 0.482±0.003 | 0.999 |
|  | 45 | 0.413±0.005 | 0.499±0.002 | 0.999 |
| KCl | 0 M | 0.968±0.009 | 0.494±0.002 | 0.999 |
|  | 0.1M | 0.918±0.010 | 0.473±0.002 | 0.999 |
|  | 0.3M | 0.729±0.005 | 0.509±0.002 | 0.999 |
|  | 0.4M | 0.453±0.003 | 0.597±0.001 | 0.999 |
| CaCl_2_ | 0 M | 1.139±0.028 | 0.454±0.005 | 0.997 |
|  | 0.1M | 1.385±0.019 | 0.425±0.003 | 0.999 |
|  | 0.3M | 1.100±0.026 | 0.447±0.005 | 0.997 |
|  | 0.4M | 1.046±0.032 | 0.451±0.007 | 0.996 |
